# Safety evaluation of recombinant Newcastle disease virus expressing IBV multi-epitope chimeric live vaccine

**DOI:** 10.3389/fmicb.2024.1458252

**Published:** 2024-07-31

**Authors:** Lei Tan, Xusheng Qiu, Lujing Liang, Xin Liao, Fei Wang, Yingjie Sun, Cuiping Song, Ying Liao, Chan Ding

**Affiliations:** Department of Avian Diseases, Shanghai Veterinary Research Institute, Chinese Academy of Agricultural Sciences, Shanghai, China

**Keywords:** Newcastle disease virus vector, infectious bronchitis virus, multiple epitopes, recombinant live vaccine, safety experiment

## Abstract

Newcastle Disease (ND) and Infectious Bronchitis (IB) are two significant diseases that pose threats to the poultry industry, caused by Newcastle disease virus (NDV) and Infectious bronchitis virus (IBV), respectively. Currently, the control and prevention of these diseases primarily rely on vaccination. However, commercial ND and IB vaccines face challenges such as poor cross-protection of inactivated IBV strains and interference from live vaccines when used together, leading to immunization failures. Previously, we reported the successful rescue of a recombinant NDV expressing multiple epitopes of IBV, named rNDV-IBV-T/B, which showed promising immunoprotective efficacy against both NDV and IBV. This study focuses on the biosafety of the genetically modified recombinant vaccine candidate rNDV-IBV-T/B. Immunization was performed on day-old chicks, ducklings, goslings, and ICR mice. Observations were recorded on clinical symptoms, body weight changes, and post-mortem examination of organs, as well as histopathological preparations of tissue samples. The results indicated that the rNDV-IBV-T/B vaccine candidate had no adverse effects on the growth of targeted animals (chickens) and non-target species (ducks, geese) as well as in mammals (mice). Additionally, histopathological slides confirmed that the vaccine is safe for all tested species. Further studies evaluated the potential of rNDV-IBV-T/B to spread horizontally and vertically post-immunization, and its environmental safety. The findings revealed that the vaccine candidate lacks the capability for both horizontal and vertical transmission and does not survive in the environment. In conclusion, the rNDV-IBV-T/B strain is safe and holds potential as a new chimeric live vaccine for ND and IB.

## 1 Introduction

Newcastle Disease (ND) and Infectious Bronchitis (IB) are two pivotal diseases in the poultry industry, causing severe economic losses globally (Zhao et al., 2017; Ike et al., 2021). ND is induced by the Newcastle disease virus (NDV), a highly contagious virus that infects various bird species. This disease manifests in diverse clinical symptoms, including respiratory, neurological, and gastrointestinal disturbances (Hu et al., 2022). The rapid spread of NDV and its extensive genetic and antigenic diversity pose significant challenges to vaccine development and disease management. Infectious Bronchitis is caused by the Infectious bronchitis virus (IBV), which predominantly infects chickens, leading to respiratory illness. In laying hens, IBV not only reduces egg production but also affects egg quality (Najimudeen et al., 2020; Farooq et al., 2023). Unlike NDV, IBV evolves rapidly, resulting in multiple serotypes that lack cross-protection, thereby complicating vaccine efficacy due to its genetic diversity (Rohaim et al., 2020). The severity and economic impact of IB can be exacerbated by co-infections with other pathogens, such as avian influenza and Mycoplasma, further complicating disease management in poultry operations (Samy and Naguib, 2018).

Currently approved vaccines for the prevention and control of ND and IB are predominantly bivalent inactivated vaccines or monovalent forms (Abozeid et al., 2019; Li et al., 2023). Inactivated ND-IB vaccines present several drawbacks. They necessitate a withdrawal period before vaccinated poultry can be processed for human consumption and require individual administration via subcutaneous or intramuscular injection (Dimitrov et al., 2017). Additionally, these vaccines do not elicit cellular immunity, demand large doses and adjuvants, and involve complex preparation processes, leading to high production costs. Even whole-virus inactivated vaccines require multiple booster immunizations to generate sufficient neutralizing antibodies.

Live attenuated viral IB vaccines have several limitations, including the potential for reversion to virulence and tissue damage. Tissue damage caused by live vaccines can lead to pathological disorders or secondary bacterial infections, particularly in day-old chicks. For instance, H52 and H120 IBV vaccines have been shown to induce significant pathology in the trachea. Additionally, potential recombination between vaccine strains and virulent field strains may result in the emergence of new IBV serotypes (Ennaji et al., 2020). Moreover, the numerous serotypes of IBV exhibit poor cross-protection. A vaccine derived from a single serotype, whether live attenuated or inactivated, provides protection only against the same serotype and offers limited or no protection against other serotypes of the virus (Keep et al., 2020).

To address the limitations of existing ND-IB vaccines, we conducted a comprehensive screening of S1 T lymphocyte and B lymphocyte epitopes from various IBV subtypes. The identified functional epitope clusters were incorporated into a DNA vaccine vector, resulting in a recombinant IBV multi-epitope vaccine with robust immunogenicity against IBV challenge (Tan et al., 2016a,b). Building on this, we employed an NDV vector and inserted the validated IBV epitope cluster between the P and M genes of NDV, creating a recombinant NDV expressing the IBV multi-epitope as a novel candidate ND-IB vaccine, designated as rNDV-IBV-T/B. Immunization of chicks with this recombinant vaccine conferred protection against lethal doses of both NDV and IBV, demonstrating its potential to simultaneously prevent NDV and IBV challenges (Tan et al., 2019). This vaccine candidate exhibits high viral titers, genetic stability, strong immunogenicity, and a long duration of immune protection.

Moreover, the production costs of this chimeric bivalent live vaccine rNDV-IBV-T/B are significantly lower than those of the conventional ND-IBV bivalent inactivated and live attenuated vaccines. The immunization route is via nasal drops or eye drops, which is more time-efficient and less labor-intensive compared to inactivated vaccines. Besides this vaccine reduces the stress on the body caused by multiple immunizations and plays a crucial role in enhancing the body’s immunity and effectively blocking pathogen invasion. It improves the productivity and quality of poultry output, which contributes positively to stabilizing the national economy and enhancing export trade, yielding significant societal benefits. Compared to single vaccines for ND and IB, the candidate vaccine strain rNDV-IBV-T/B reduces the number of immunizations required and effectively controls the prevalence and incidence of these two respiratory infectious diseases.

In order to evaluate the safety of the candidate vaccine strain rNDV-IBV-T/B, this study conducted a biosafety assessment of the recombinant virus vaccine, encompassing several aspects. Briefly, the evaluation involved assessing clinical symptoms, weight gain, organ dissection, and histopathological slices of vaccinated target animals (chickens) and non-target animals (ducks, geese, and mice). Subsequently, the proliferation, distribution, and shedding of the rNDV-IBV-T/B in vaccinated chickens were assessed, along with its capability for horizontal and vertical transmission. Furthermore, the survival ability of the recombinant NDV in environmental applications was also evaluated.

The results indicate that the presence of the rNDV-IBV-T/B in immunized chickens does not induce clinical symptoms or pathological changes associated with NDV, demonstrating good biosafety for the target animals. Additionally, the rNDV-IBV-T/B strain lacks the ability to horizontally transmit to cohabiting non-immunized chickens and does not have the capability to transmit vertically to progeny. Overall, these findings suggest that rNDV-IBV-T/B is safe and holds potential as a novel chimeric live prophylactic vaccine for ND-IB.

## 2 Materials and methods

### 2.1 Ethics declarations

The authors confirm that the ethical policies of the journal, as noted on the journal’s author guidelines page have been adhered to. The Ethics and Animal Welfare Committee of Shanghai Veterinary Research Institute, China reviewed the all experiments procedures and approval this project (SV-20230728-Y01). The study was carried out in compliance with Guidelines for the Euthanasia of Animals.

### 2.2 Animal, viruses, vaccine, reagent

Fertile white leghorn SPF embryonated eggs (Beijing Boehringer Ingelheim Vital Biotechnology Co, Ltd. Beijing, China) were used for testing the 50% embryo infectious dose (EID_50_). ICR mice were purchased from Shanghai Sippe-Bk Lab Animal Co, Ltd. One-day-old duck and gosling were purchased from Zhejiang Lihua Agricultural Technology Co., Ltd. The recombinant NDV express IBV multiple epitopes vaccine candidate strain rNDV-IBV-T/B were rescued based on the reverse genetic system in our lab and reported previously (Tan et al., 2019). The avirulent NDV LaSota vaccine strain (Batch No. 150132007) is product of Qingdao Yebio Biological Engineering Co Ltd. Reverse transcription kit GoScript^™^ Reverse Transcriptase was purchased from Promega (Catalog number: A2791). TRIzol reagent (Catalog number: 15596018, Invitrogen). DL2000 Plus DNA Marker (Catalog number: MD101-01), Ultra GelRed Nucleic Acid Dye (Catalog number: GR501-01) and Phanta Super-Fidelity DNA Polymerase (Catalog number: P501-d1) are products of Nanjing Vazyme Biotech Co Ltd.

### 2.3 Virus titration

The virus titration of the rNDV-IBV-T/B were carried out according to a previously reported method (Grimes, 2002). The hemagglutination test (HA) assay, EID_50_ assay were performed in 96-well micro-plates, 10 day-old SPF chicken embryos, respectively, to determine the infectivity titration of the recombinant NDV rNDV-IBV-T/B strain. The numeric value of EID_50_ were then calculated according to the Reed–Muench reported method (Reed and Muench, 1938).

### 2.4 RNA preparation

Collect viral samples and transfer them into eppendorf (EP) centrifuge tubes. Add TRIzol reagent (1 mL per 100 μL of sample), mix thoroughly, and incubate at room temperature for 5 min. Add 200 μL of chloroform per 1 mL of TRIzol, cap the tubes tightly, shake vigorously for 15 s, and incubate at room temperature for 2–3 min. Centrifuge at 12,000 × g for 15 min at 4°C. Transfer the upper aqueous phase to a new tube, add 500 μL of isopropanol per 1 mL of TRIzol, mix by inversion, and incubate at room temperature for 10 min. Centrifuge at 12,000 × g for 10 min at 4°C. Discard the supernatant, wash the RNA pellet with 1 mL of 70% ethanol, and centrifuge at 7,500 × g for 5 min at 4°C. Remove ethanol, air-dry the RNA pellet, and dissolve it in RNase-free water. Incubate at 55–60°C for 10–15 min to ensure complete dissolution. For reverse transcription (RT) RNA to cDNA, prepare the RT reaction mixture on ice following the GoScript^™^ Reverse Transcriptase (Promega) instructions. A typical mixture includes: 1 μg RNA template, 1 μL Oligo(dT) primer, 1 μL dNTP mix (10 mM each), 4 μL 5X RT buffer, 1 μL RNase inhibitor, 1 μL reverse transcriptase, and RNase-free water to a final volume of 20 μL. Incubate the RNA and primer mixture at 65°C for 5 min, then place on ice. Add remaining components, mix gently, and incubate at 42°C for 30 min. Terminate the reaction by heating at 70–85°C for 5–10 min. The synthesized cDNA can be used immediately or stored at −20°C for long-term storage.

### 2.5 PCR assay

Utilizing the extracted cDNA from the recombinant virus vaccine and Phanta Super-Fidelity DNA Polymerase, NDV P gene-specific primers were employed for amplification: F-5’ ATGGGCYCCAGAYCTTCTAC-3′ and R-5’ CTGCCACTGCTA GTTGTGATAATCC-3′. The target fragment is approximately 535 bp. The PCR program was set on a thermocycler as follows: initial denaturation at 95°C for 3 min, followed by 30 cycles of denaturation at 95°C for 30 s, annealing at 58°C for 45 s, and extension at 72°C for 30 s, with a final extension at 72°C for 8 min. The PCR amplification products were analyzed by 1% agarose gel electrophoresis. A DNA DL2000 marker was used as a reference. The electrophoresis was performed at 120 V for 30 min, and the results were recorded using a gel imaging system.

### 2.6 Histopathological analysis

Sterile samples were collected from the major organs and tissues of immunized chickens, including the heart, liver, spleen, lungs, kidneys, trachea, and bursa of Fabricius et al. These samples were cut into small pieces and fixed in 10% neutral buffered formalin for 48 h. After fixation, the tissues were dehydrated in ethanol solutions (70, 80, 90, 95, and 100%), cleared in xylene for 30 min, and embedded in paraffin. The paraffin-embedded tissues were then sectioned into 3–5 micron slices using a microtome. The slices were floated on a warm water bath, transferred onto glass slides, and dried in an oven at 60°C for 30–60 min. The sections were stained with Hematoxylin and Eosin (H&E). The slides were deparaffinized in xylene, rehydrated through graded ethanol (100, 95, 70%), and rinsed in tap water. They were then stained with hematoxylin, differentiated in 1% hydrochloric acid ethanol, rinsed, blued in 0.5% ammonia water, counterstained with eosin, and rinsed again. After staining, the sections were dehydrated through ascending ethanol, cleared in xylene, and mounted with cover slips to remove air bubbles.

### 2.7 Statistical analysis

The data were analyzed using one-way ANOVA through GraphPad Prism v8.02 (San Diego, CA, United States) and the results were expressed as the mean ± standard error of the mean (SEM). A probability value (*P*) of <0.05 was considered to be significant.

## 3 Results

### 3.1 Effects of rNDV-IBV-T/B strains on weight gain in chickens

**Figure 1:**
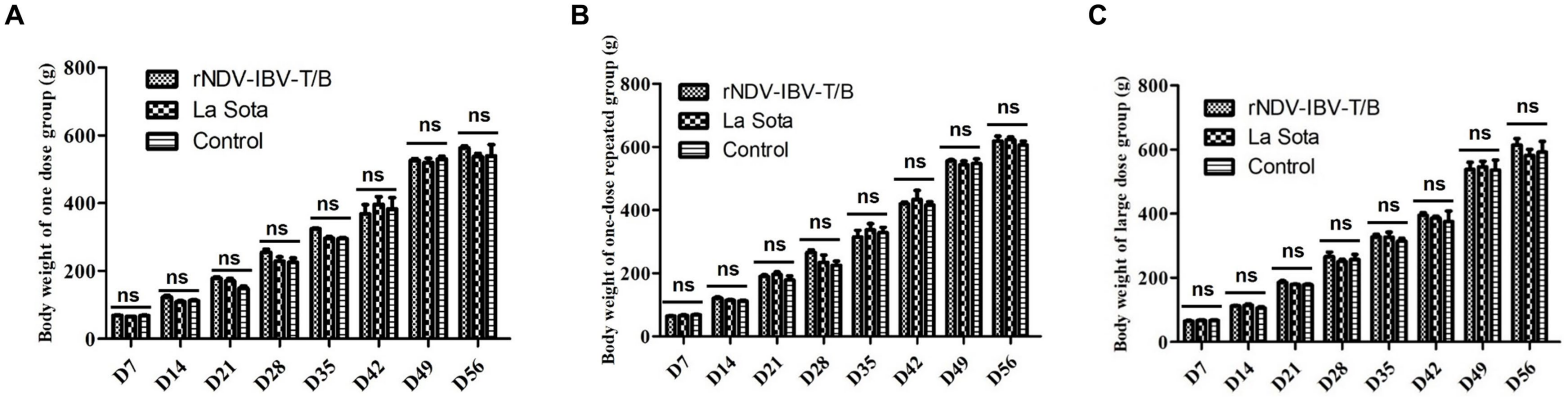
Body weight index in chickens immunized with rNDV-IBV-T/B strains in different immune schemes. **(A)** Single dose group (10^6^ EID50/chick); **(B)** single-dose repeated group (Prime with 10^6^ EID50 per chick on day 1, followed by a booster of 10^6^ EID50 per chick on day 14); **(C)** large-dose group (10^8^ EID50/chick). The chicks in the vaccine-immunized group and the control group were weighed every 7 days and observed until 56 days. Error bars represent ± SEM (standard error of the mean), and “ns” indicates *p* > 0.05.

To evaluate the impact of different doses and immunization protocols of the rNDV-IBV-T/B on weight gain in target animals (chickens), we conducted a study with three immunization protocols (Table 1). The first protocol was a single-dose vaccination group, the second was a repeated single-dose vaccination group, and the third was a high-dose single vaccination group. Each immunization protocol involved randomly selecting 45 one-day-old SPF chicks and dividing them into the rNDV-IBV-T/B immunization group, the NDV commercial vaccine LaSota strain immunization group, and the sterile PBS control group. In the single-dose vaccination trial, the rNDV-IBV-T/B and LaSota vaccine groups were immunized via intranasal and ocular routes with 10^6^ EID_50_/200 μL per chick, while the control group received sterile PBS, 200 μL per chick. In the repeated single-dose vaccination trial, an initial immunization of 10^6^ EID_50_/200 μL per chick was followed by a booster of the same dose 14 days later. In the high-dose single vaccination trial, chicks were immunized with 10^8^ EID_50_/200 μL per chick. Post-immunization, the chicks were housed in separate isolators and monitored daily for mental state, feed and water intake, fever, diarrhea, morbidity, and mortality. Weighing of the chicks in both the vaccine and control groups was conducted every 7 days until day 56. The results indicated that the chickens in the immunized groups exhibited good mental state and normal feeding and drinking behavior, with no morbidity or mortality. There were no significant differences in weight gain among the rNDV-IBV-T/B, LaSota, and PBS control groups during the observation period (*p* > 0.05), demonstrating the good safety profile of rNDV-IBV-T/B across different doses and immunization protocols, as shown in Figures 1A–C.

**Table 1:** Immunization protocols for chickens.

Group	Vaccine strain	Numbers	Dose of inoculation	Route of administration	Prime	Boost
A1	rNDV-IBV-T/B	15	10^6^EID_50_/200 μL	Intranasal	1	/
A2	LaSota	15	10^6^EID_50_/200 μL	Intranasal	1	/
A3	PBS	15	200 μL	Intranasal	1	/
B1	rNDV-IBV-T/B	15	10^6^EID_50_/200 μL	Intranasal	1	14
B2	LaSota	15	10^6^EID_50_/200 μL	Intranasal	1	14
B3	PBS	15	200 μL	Intranasal	1	/
C1	rNDV-IBV-T/B	15	10^8^EID_50_/200 μL	Intranasal	1	/
C2	LaSota	15	10^8^EID_50_/200 μL	Intranasal	1	/
C3	PBS	15	200 μL	Intranasal	1	/

### 3.2 Evaluation of the effect of rNDV-IBV-T/B on weight gain in ducks, geese and mice

**Figure 2:**
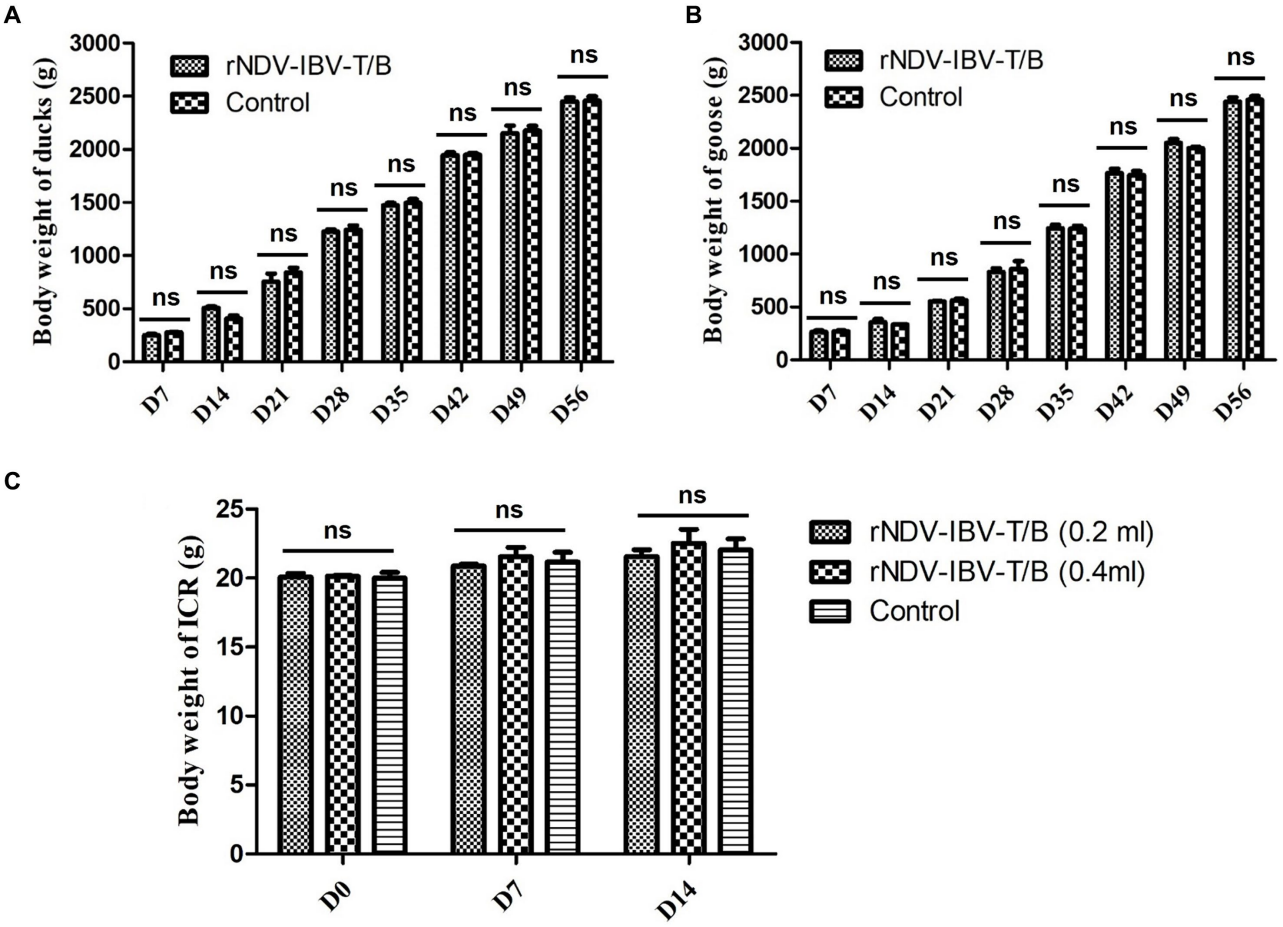
Monitoring weight changes in non-target animals immunized with the candidate vaccine strain rNDV-IBV-T/B. **(A)** ducks, **(B)** geese and **(C)** mice were in the rNDV-IBV-T/B immunized group and the control group were weighed every 7 days. The ducks and geese were monitored for 56 days following immunization, while the mice were monitored for 14 days post-immunization due to slight changes in body weight. ns represents *p* > 0.05.

In this study, we focused on evaluating the impact of the rNDV-IBV-T/B on weight gain in non-target waterfowl species (ducklings and goslings) and mammals (mice). Waterfowl immunization groups and protocols: Ducklings and goslings were divided into an rNDV-IBV-T/B immunization group and a PBS control group, with 15 animals in each group. The immunization dose was 10^6^ EID_50_/200 μL. Post-immunization, the animals were monitored daily for health status, and their weight was recorded weekly until day 56. Mammalian Mice immunization protocol: Mice were divided into three groups: a single-dose rNDV-IBV-T/B immunization group (10^6^ EID_50_/200 μL per mouse), a double-dose immunization group (2*10^6^ EID_50_/400 μL per mouse), and a PBS control group. Due to the slower weight gain in mice, the observation period was limited to 14 days. Statistical methods were employed to compare weight gain differences between the rNDV-IBV-T/B immunization groups and the PBS control groups in ducklings, goslings, and mice. The results indicated that during the observation period, the rNDV-IBV-T/B immunized ducklings, goslings, and mice exhibited good growth status with no adverse reactions. There were no significant differences in weight gain compared to the PBS control groups (*p* > 0.05), as shown in Figures 2A–C.

### 3.3 Histopathological lesions analysis in rNDV-IBV-T/B strain immunized chickens

**Figure 3:**
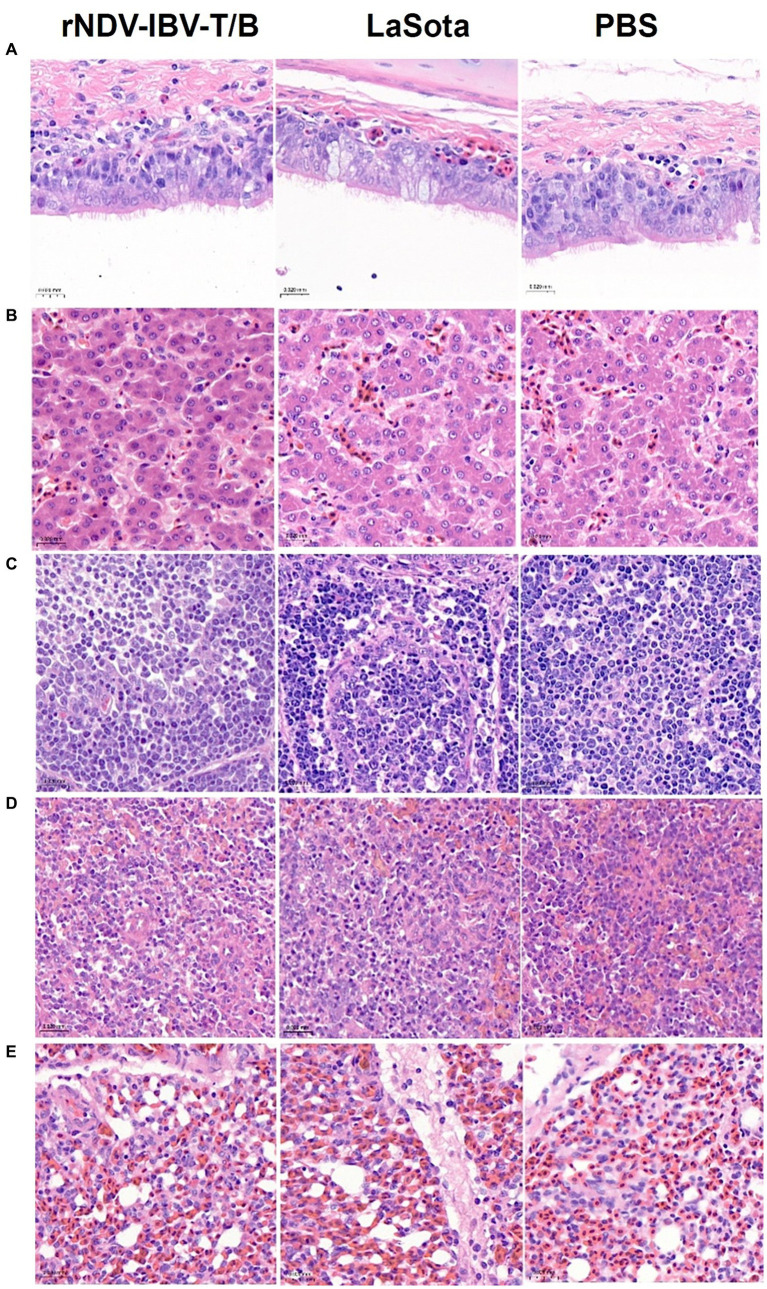
Histopathological lesions analysis. Sections of **(A)**: trachea, **(B)**: liver, **(C)**: bursa of Fabricius, **(D)**: spleen, and **(E)**: lungs from the rNDV-IBV-T/B immunized group, LaSota immunized group, and PBS group. H&E stain. Scale bars = 20 μm.

To evaluate the impact of the rNDV-IBV-T/B on the growth, development, and pathology of major organs in target animals (chickens), we established three groups: the rNDV-IBV-T/B immunization group (10^6^ EID_50_/200 μL/chicken), the LaSota vaccine immunization group, and the PBS control group, with 10 chickens in each group. Thirty-five days post-immunization, the chickens were euthanized, and sterile dissections were performed to collect tissues from the trachea, liver, bursa of Fabricius, spleen, and lungs. Pathological sections were prepared and observed using a panoramic scanner (3D HISTECH Pannoramic MIDI, MADE IN Hungary). Pathological changes were evaluated based on the severity of lesions, ranging from mild to severe. No lesions were scored as 0 (negative), slight lesions as 1, mild lesions as 2, moderate lesions as 3, and severe lesions as 4. The results showed that during the observation period, the tissue structures in the rNDV-IBV-T/B immunization group were intact and clear, with no significant necrosis or swelling. There were no significant pathological differences between the rNDV-IBV-T/B immunized group and the LaSota vaccine immunized group or the PBS control group (*p* > 0.05), as shown in Figures 3A–E.

### 3.4 Evaluation of the effects of rNDV-IBV-T/B strain immunization on organ growth and pathological changes in ducks, geese and mice

**Figure 4:**
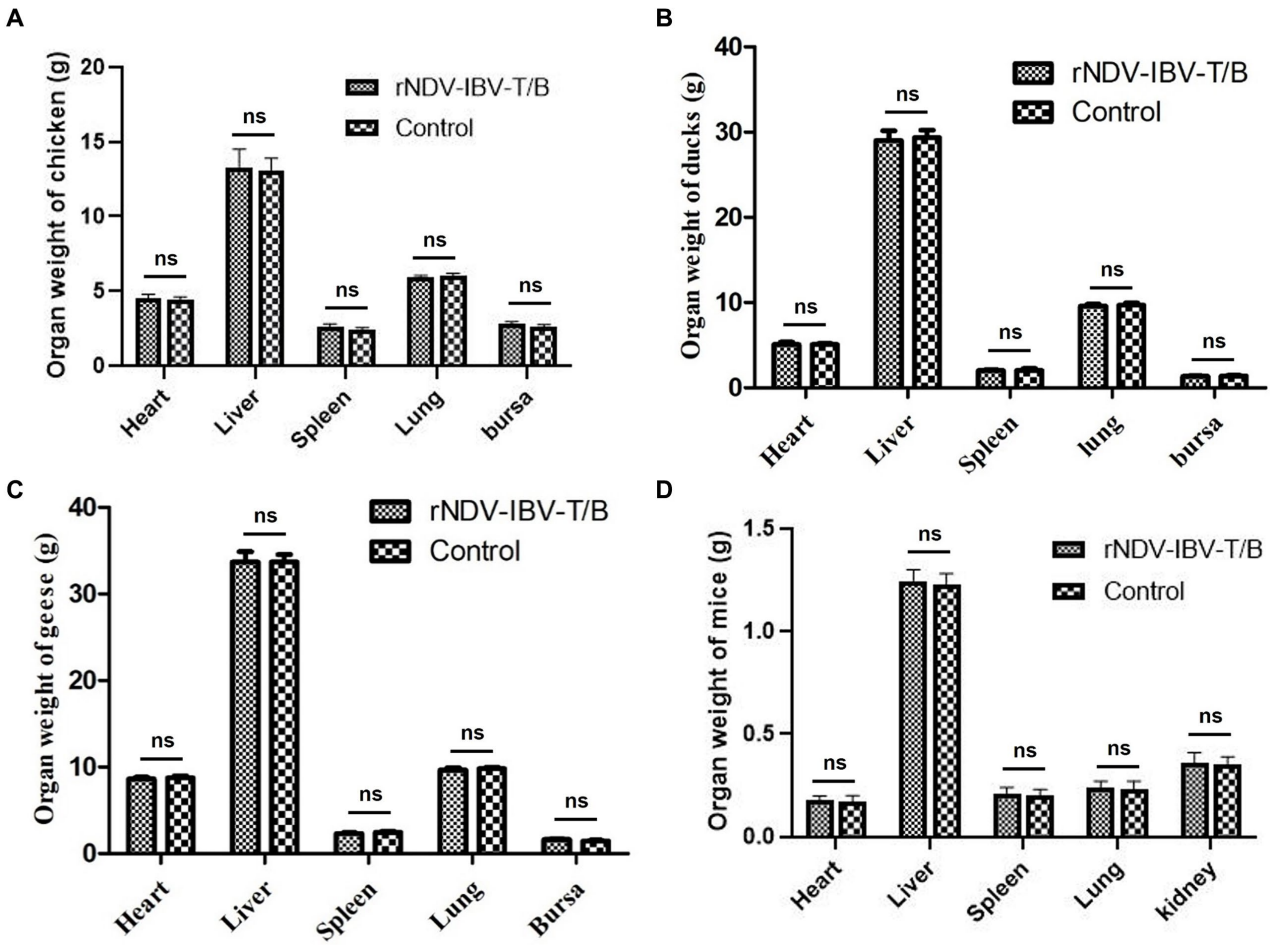
Analysis of organ weight index changes. Effects of rNDV-IBV-T/B strain immunization on the growth and development of tissues and organs in target animal **(A)** chicken and non-target animals **(B)** ducks, **(C)** geese **(D)** mice. The organs were weighed and the statistical differences between the rNDV-IBV-T/B immunized group and the PBS control group were analyzed. Error bars represent ± SEM and ns indicates *p* > 0.05.

To evaluate the impact of the rNDV-IBV-T/B on the growth and development of major organs in target chickens and non-target animals (ducklings, goslings, and mice), we established experimental groups for 1 day-old chicks, ducklings, goslings, and 3 week-old mice. Each species was divided into an rNDV-IBV-T/B immunization group (10^6^ EID_50_/200 μL per individual) and a PBS control group, with 10 individuals per group. At 35 days post-immunization, the animals were euthanized and necropsied. Major organs, including the heart, liver, spleen, lungs, and bursa of Fabricius, were aseptically collected, weighed, and recorded. Statistical methods were used to compare the weight variations of organs between the rNDV-IBV-T/B and PBS control groups. The results showed no significant differences (*p* > 0.05) in the weights of the heart, liver, spleen, lungs, and bursa of Fabricius among different immunization groups for chicks, ducklings, and goslings (Figures 4A–C). Additionally, the weights of the heart, liver, spleen, lungs, and kidneys in mice were analyzed. The results indicated no significant differences (*p* > 0.05) in these organ weights between the rNDV-IBV-T/B immunization group and the PBS control group (Figure 4D). These findings suggest that the candidate vaccine strain rNDV-IBV-T/B does not adversely affect the growth and development of major organs in immunized animals, demonstrating good safety.

### 3.5 Evaluation of the horizontal transmission ability of rNDV-IBV-T/B strain in immunized chickens

**Figure 5:**
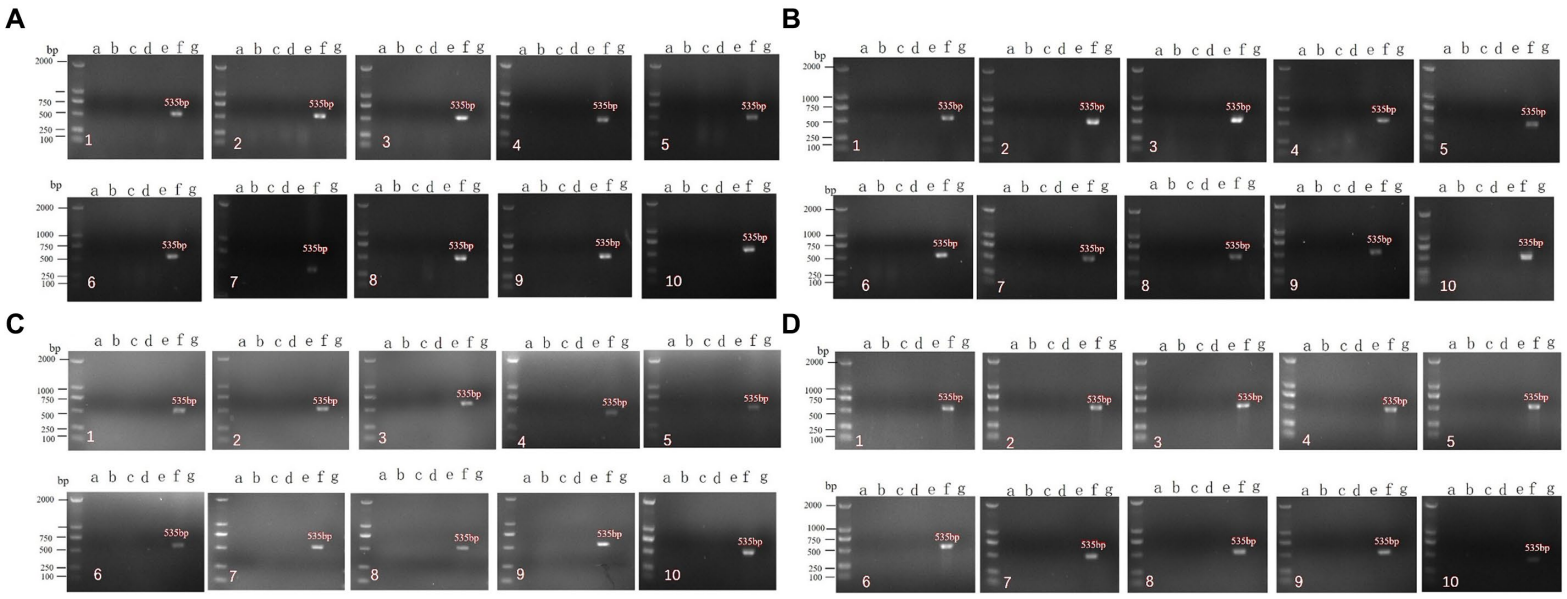
PCR detection of horizontal transmission ability post-immunization with the vaccine candidate strain rNDV-IBV-T/B. Panels **(A–D)** represent samples collected on days 7, 14, 21, and 28, respectively. a–g represent the following groups: a: rNDV-IBV-T/B immunized group; b: rNDV-IBV-T/B co-housed non-immunized group; c: La Sota immunized group; d: La Sota co-housed non-immunized group; e: PBS control group; f: NDV positive control; g: negative control. 1–10 represent the different organs and samples: 1: heart; 2: liver; 3: spleen; 4: lungs; 5: kidneys; 6: cecum; 7: bursa of Fabricius; 8: glandular stomach; 9: tracheal swab; 10: cloacal swab. DL2000 is used as DNA Marker.

After immunization with the rNDV-IBV-T/B, chickens may potentially transmit the virus to co-housed target animals through air or contact. This experiment aimed to evaluate the horizontal transmission capability of this vaccine to non-immunized chickens in the same enclosure. Ninety 1 day-old chicks were randomly divided into three groups: the rNDV-IBV-T/B immunization group, the LaSota immunization group, and the PBS control group, with 30 chickens in each group. Fifteen chickens in each group were randomly immunized via intranasal and ocular routes at a dose of 10^6^ EID_50_/0.2 mL per chick, while the remaining 15 chickens were not immunized but were housed in the same isolator. On days 7, 14, 21, and 28 post-immunization, sterile samples were collected from the heart, liver, spleen, kidneys, lungs, cecum, bursa of Fabricius, and glandular stomach of both immunized and co-housed non-immunized chickens. Throat and cloacal swabs were also collected. Viral RNA was extracted, and PCR detection was performed using NDV-specific primers. The distribution of the rNDV-IBV-T/B strain in various organs, viral shedding, and horizontal transmission capability to co-housed chickens were analyzed. The specific NDV gene size is 535 bp. The results showed that no NDV target gene was amplified from samples collected from either immunized or co-housed chickens (Figures 5A–D). This indicates that the vaccine candidate strain rNDV-IBV-T/B does not have the ability for horizontal transmission post-immunization.

### 3.6 Vertical transmission ability of rNDV-IBV-T/B strain in immunized chickens

**Figure 6:**
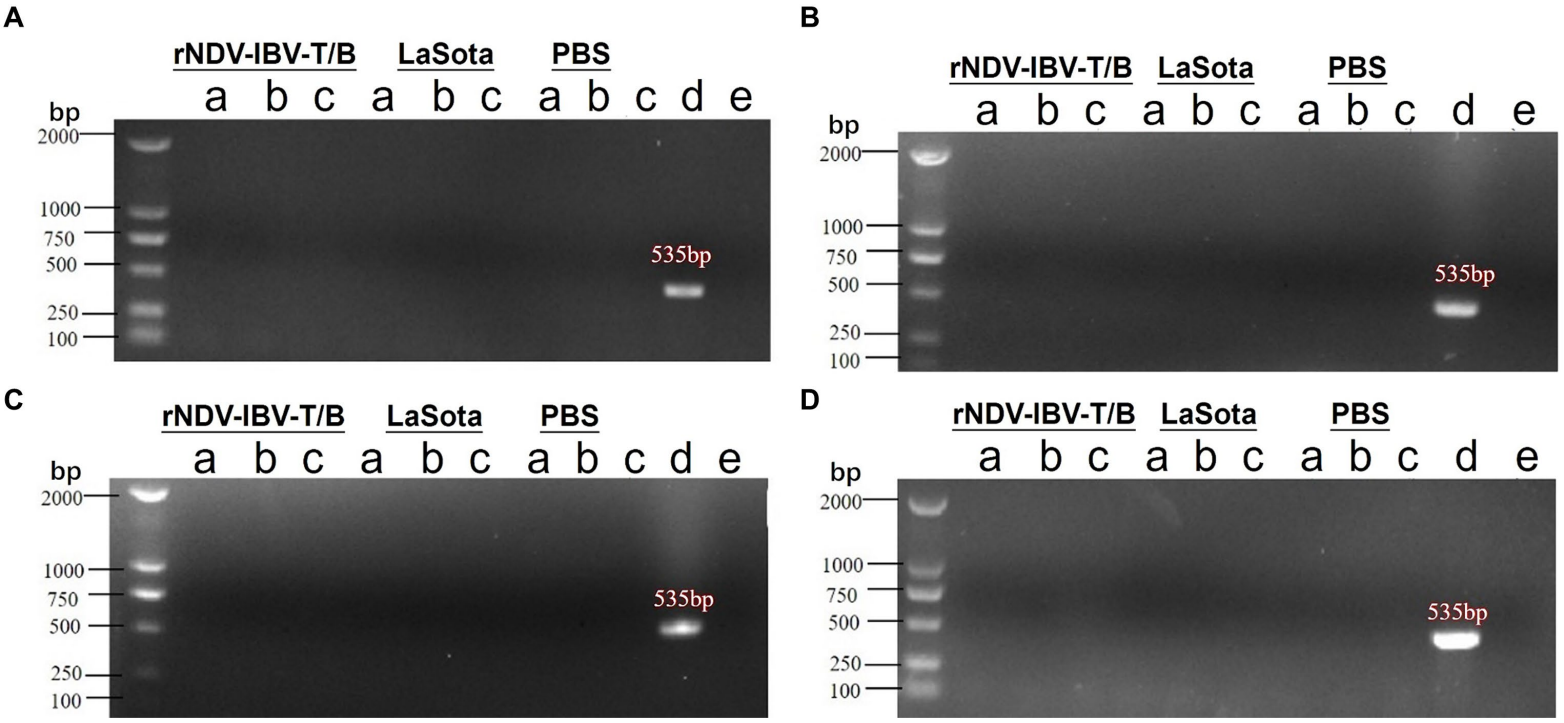
PCR detection of vertical transmission ability post-immunization with the candidate vaccine rNDV-IBV-T/B. Laying hens were immunized with the rNDV-IBV-T/B vaccine, LaSota vaccine, and PBS control, respectively. Samples were aseptically collected from the **(A)** oviduct, **(B)** ovary, and **(C)** egg yolk on days **(A)** 7, **(B)** 14, **(C)** 21, and **(D)** 28 after the onset of egg production in each group. Viral RNA was extracted, and PCR detection was performed. Positive (d) and negative **(E)** controls were also included for each group. DL2000 is used as DNA Marker.

After immunization with the rNDV-IBV-T/B, laying hens may potentially transmit the virus vertically through the oviduct to infect progeny embryos. To evaluate the vertical transmission capability of this vaccine post-immunization in laying hens, the following experiment was conducted. Ninety 1 day-old laying hens were randomly divided into three groups, with 30 chickens in each group. Each group was immunized with either rNDV-IBV-T/B, La Sota, or PBS control via intranasal and ocular routes at a dose of 10^6^ EID_50_/0.2 mL per chicken. On days 7, 14, 21, and 28 after the onset of egg production, five chickens from each group were randomly selected, and samples were aseptically collected from the oviduct, ovary, and egg yolk. RNA was extracted from the samples, and PCR amplification of the cDNA was performed using NDV-specific primers. Negative and positive controls were included, and agarose gel electrophoresis was conducted for nucleic acid detection. The electrophoresis results showed that at various time points, no NDV-specific genes were amplified from the samples collected from the rNDV-IBV-T/B immunized group, as shown in Figures 6A–E. This indicates that the vaccine candidate strain rNDV-IBV-T/B does not possess vertical transmission capability in immunized chickens.

### 3.7 Virus particle shedding of rNDV-IBV-T/B in immunized chickens and its impact on the environment

**Figure 7:**
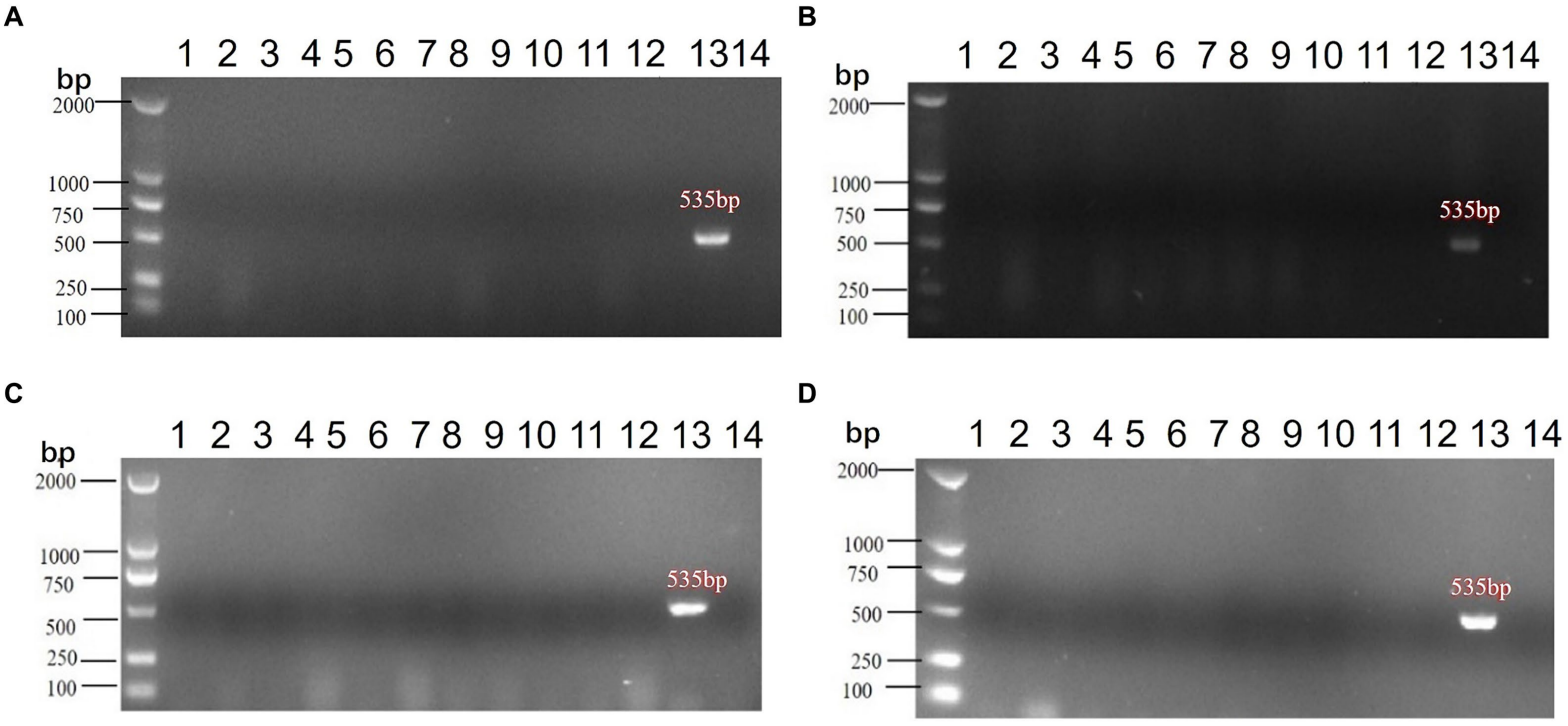
PCR detection of environmental impact post-immunization with the rNDV-IBV-T/B strain. Samples from **(A)** 3d, **(B)** 7d, **(C)** 14d, **(D)** 21d were collected post-immunization for PCR testing, with controls included. The samples collected were as follows: (1) Tracheal swabs, (2) Cloacal swabs, (3) Feces, (4) Feeding devices, (5) Drinking water containers, (6) Bedding material, (7) Air filters, (8) Dust particles, (9) Soil samples, (10) Surrounding water sources, (11) Neighboring enclosures, (12) Caretaker clothing&Boots, (13) Positive control, (14) Negative control. DL2000 is used as DNA Marker.

Following vaccination with a live vaccine, animals may shed viral particles through the cloaca, potentially releasing them into the environment and posing an environmental impact. This study evaluates the shedding of the candidate vaccine strain rNDV-IBV-T/B post-immunization and its potential environmental impact. Thirty 1 day-old chicks were immunized via intranasal and ocular routes with a dose of 10^6^ EID_50_/0.2 mL per chick. On days 3, 7, 14, and 21 post-immunization, three chickens were randomly selected and euthanized. Sterile samples were collected, including tracheal swabs and cloacal swabs from the chickens. Environmental samples were also collected, encompassing feces, feeding devices, drinking water containers, bedding material, air filters, dust particles, soil samples, neighboring animal enclosures, and personnel equipment such as clothing and boots used by handlers. Viral RNA was extracted from all samples, and PCR amplification was performed using NDV-specific primers. The results indicated that no NDV target fragments were detected in any of the samples at all time points, as shown in Figures 7A–D. This suggests that the candidate vaccine strain rNDV-IBV-T/B does not shed viral particles post-immunization and thus poses no environmental impact.

## 4 Discussion

In our previous studies, we successfully constructed the recombinant vaccine candidate strain rNDV-IBV-T/B and evaluated its biological characteristics, genetic stability, and immunoprotective efficacy. The results demonstrated that this vaccine meets the standards for NDV attenuated vaccines. After multiple passages in chicken embryos, the expressed IBV S1 protein, encompassing various T and B lymphocyte epitopes, showed good genetic stability. Immunoprotection experiments confirmed that the vaccine provided 90 and 100% survival protection rates against lethal challenges with IBV and NDV, respectively, indicating that the rNDV-IBV-T/B candidate vaccine possesses good immunogenicity. Furthermore, We have continuously passaged the vaccine candidate rNDV-IBV-T/B for 25 times, and gene sequencing has shown that the protective antigen epitope box of IBV has not mutated or been deleted, and has good genetic stability (Tan et al., 2019). These findings align with previous reports on recombinant NDV-based bivalent vaccines expressing the IBV S protein. However, those vaccines primarily express the IBV S protein, S1 protein, or S2 protein, which may provide effective protection against a single IBV subtype but may not ensure reliable cross-protection against different IBV subtypes (Eldemery et al., 2017; Zhao et al., 2017; Shirvani et al., 2018). Thus, there is a critical need to develop novel vaccines that can protect against multiple IBV subtypes.

To further advance the registration of the recombinant vaccine candidate strain rNDV-IBV-T/B as a veterinary biological product, this study evaluated its transgenic safety in accordance with the experimental requirements stipulated by the “Regulations on the Biosafety Evaluation of Agricultural Genetically Modified Organisms” of the Ministry of Agriculture and Rural Affairs of China. The evaluation primarily focused on the safety of the vaccine candidate strain rNDV-IBV-T/B in both target and non-target animals. Additionally, the study investigated the horizontal and vertical transmission capabilities of the vaccine, as well as its survival in the environment.

In the study, target chickens were immunized with the candidate vaccine strain rNDV-IBV-T/B, with experimental groups set up for single-dose immunization, single-dose repeated immunization, and large-dose administrations. Each experimental group was paralleled by control groups using the parent strain commercial LaSota vaccine and PBS. Clinical observations post-immunization included the animals’ mental state, feed and water intake, weight changes, and variations in the weights of various organs. Biostatistical analysis indicated that there were no significant differences (*p* > 0.05) in feed and water intake, growth, and weight gain between the rNDV-IBV-T/B immunized group, the LaSota immunized group, and the PBS control group at any time point.

Additionally, pathological analysis of major organs, including the heart, liver, spleen, lungs, and bursa of Fabricius, in immunized target chickens showed no observable changes in organ tissues. The histopathological examination revealed intact and clear structures without any pathological lesions. This indicates that the vaccine candidate strain rNDV-IBV-T/B is non-pathogenic and has a good safety profile when administered to immunized animals.

Furthermore, to evaluate the safety of the candidate vaccine strain rNDV-IBV-T/B in non-target waterfowl species such as ducklings and goslings, as well as in immunized mammalian mice, we conducted relevant studies. The results indicated normal growth and weight gain post-immunization, with no pathogenic effects on visceral organs. Histopathological results confirmed the good safety profile of the vaccine in immunized non-target animals. These findings are consistent with previously reported safety evaluations of recombinant live vaccines using NDV as a vector to express various exogenous genes (Kim and Samal, 2016; Lara-Puente et al., 2021; Lee et al., 2021; Tcheou et al., 2021). This further corroborates the safety of the NDV LaSota strain as a viral vector.

Previous studies have shown that IBV live vaccine can spread horizontally through aerosols among different chickens (Laconi et al., 2020; Falchieri et al., 2024). If the vaccine virus spreads horizontally within a flock, it can compensate for any missed immunizations, thereby enhancing the overall immunity of the flock. However, the prolonged presence of the vaccine virus in the flock may lead to a reversion to virulence. Furthermore, Pereira et al. found that IBV live vaccine could be horizontally and vertically transmitted post-immunization. The virus was excreted and transmitted to co-housed non-immunized chickens through feces. In vaccinated laying hens, IBV was detected in both eggs and 1 day-old chicks, with the S1 gene sequence identical to that of the vaccine strain, indicating the vertical transmission capability of IBV live vaccines (Pereira et al., 2016). These studies suggest that traditional IBV live vaccines still pose a potential risk of reversion to virulence. Therefore, it is crucial to consider the potential for horizontal and vertical transmission, as well as the environmental impact, when using IBV live vaccines.

Accordingly, our evaluation of the horizontal and vertical transmission capabilities and environmental survival of the vaccine candidate strain rNDV-IBV-T/B post-immunization revealed that rNDV-IBV-T/B does not have the ability to horizontally transmit to co-housed non-immunized chickens, nor does it have the capability for vertical transmission to progeny chickens. Further analysis of viral shedding post-immunization with the rNDV-IBV-T/B strain and multifactorial environmental testing revealed that no viral particles from the candidate vaccine strain were detected in the environment, indicating that the vaccine has good environmental safety. Additionally, the NDV vector used in this study expresses epitopes of the IBV S1 protein, including multiple T cell and B cell epitopes. These epitopes are non-infectious amino acid peptides, ensuring that there is no potential risk of viral recombination, nor any environmental impact.

While our study included multiple species (chicks, ducklings, goslings, and mice), we recognize that these results might not fully extrapolate to other avian species not included in our study. The species-specific could potentially influence the safety profiles of the vaccine.

In summary, our study results indicate that the vaccine candidate strain rNDV-IBV-T/B exhibits no horizontal or vertical transmission capabilities post-immunization, demonstrating good safety for both chickens and the environment. It has the potential to become a novel bivalent chimeric vaccine for Newcastle disease and infectious bronchitis.

## Data availability statement

The original contributions presented in the study are included in the article/supplementary material, further inquiries can be directed to the corresponding authors.

## Ethics statement

The animal study was approved by the Ethics and Animal Welfare Committee of Shanghai Veterinary Research Institute, China. The study was conducted in accordance with the local legislation and institutional requirements.

## Author contributions

LT: Conceptualization, Formal analysis, Writing – original draft. XQ: Formal analysis, Writing – review & editing. LL: Methodology, Writing – review & editing. XL: Investigation, Methodology, Writing – review & editing. FW: Methodology, Writing – review & editing. YS: Methodology, Writing – review & editing. CS: Investigation, Writing – review & editing. YL: Investigation, Writing – review & editing. CD: Conceptualization, Project administration, Writing – review & editing.

## References

[ref1] Abozeid H. H. Paldurai A. Varghese B. P. Khattar S. K. Afifi M. A. Zouelfakkar S.. (2019). Development of a recombinant Newcastle disease virus-vectored vaccine for infectious bronchitis virus variant strains circulating in Egypt. Vet. Res. 50:12. doi: 10.1186/s13567-019-0631-5, PMID: 30744668 PMC6371441

[ref2] Dimitrov K. M. Afonso C. L. Yu Q. Miller P. J. (2017). Newcastle disease vaccines-a solved problem or a continuous challenge? Vet. Microbiol. 206, 126–136. doi: 10.1016/j.vetmic.2016.12.019, PMID: 28024856 PMC7131810

[ref3] Eldemery F. Li Y. Yu Q. Van Santen V. L. Toro H. (2017). Infectious bronchitis virus S2 of 4/91 expressed from recombinant virus does not protect against ark-type challenge. Avian Dis. 61, 397–401. doi: 10.1637/11632-032017-ResNoteR, PMID: 28957002

[ref4] Ennaji Y. Khataby K. Ennaji M. M. (2020). “Chapter 3—infectious bronchitis virus in poultry: molecular epidemiology and factors leading to the emergence and reemergence of novel strains of infectious bronchitis virus” in Emerging and reemerging viral pathogens. ed. Ennaji M. M. (Amsterdam, The Netherlands: Academic Press, Elsevier Inc), 31–44.

[ref5] Falchieri M. Coward V. J. Reid S. M. Lewis T. Banyard A. C. (2024). Infectious bronchitis virus: an overview of the "chicken coronavirus". J. Med. Microbiol. 73:1828. doi: 10.1099/jmm.0.001828 PMC11184965 38771617

[ref6] Farooq M. Abd-Elsalam R. M. Ratcliff N. Hassan M. S. H. Najimudeen S. M. Cork S. C.. (2023). Comparative pathogenicity of infectious bronchitis virus Massachusetts and Delmarva (DMV/1639) genotypes in laying hens. Front Vet Sci 10:1329430. doi: 10.3389/fvets.2023.1329430 38313768 PMC10834656

[ref7] Grimes S. (2002). A BASIC LABORATORY MANUAL for THE SMALL-SCALE PRODUCTION AND TESTING OF I-2 NEWCASTLE DISEASE VACCINE).

[ref8] Hu Z. He X. Deng J. Hu J. Liu X. (2022). Current situation and future direction of Newcastle disease vaccines. Vet. Res. 53:99. doi: 10.1186/s13567-022-01118-w, PMID: 36435802 PMC9701384

[ref9] Ike A. C. Ononugbo C. M. Obi O. J. Onu C. J. Olovo C. V. Muo S. O.. (2021). Towards improved use of vaccination in the control of infectious bronchitis and Newcastle disease in poultry: understanding the immunological mechanisms. Vaccines 9:10020. doi: 10.3390/vaccines9010020 PMC7823560 33406695

[ref10] Keep S. Sives S. Stevenson-Leggett P. Britton P. Vervelde L. Bickerton E. (2020). Limited cross-protection against infectious bronchitis provided by recombinant infectious bronchitis viruses expressing heterologous spike glycoproteins. Vaccines 8:20330. doi: 10.3390/vaccines8020330 PMC7350270 32580371

[ref11] Kim S. H. Samal S. K. (2016). Newcastle disease virus as a vaccine vector for development of human and veterinary vaccines. Viruses 8:70183. doi: 10.3390/v8070183, PMID: 27384578 PMC4974518

[ref12] Laconi A. Weerts E. Bloodgood J. C. G. Deniz Marrero J. P. Berends A. J. Cocciolo G.. (2020). Attenuated live infectious bronchitis virus QX vaccine disseminates slowly to target organs distant from the site of inoculation. Vaccine 38, 1486–1493. doi: 10.1016/j.vaccine.2019.11.064, PMID: 31822427 PMC7115521

[ref13] Lara-Puente J. H. Carreño J. M. Sun W. Suárez-Martínez A. Ramírez-Martínez L. Quezada-Monroy F.. (2021). Safety and immunogenicity of a Newcastle disease virus vector-based SARS-CoV-2 vaccine candidate, AVX/COVID-12-HEXAPRO (patria), in pigs. MBio 12:e0190821. doi: 10.1128/mBio.01908-21 34544278 PMC8546847

[ref14] Lee J. Kim D. H. Noh J. Youk S. Jeong J. H. Lee J. B.. (2021). Live recombinant NDV-vectored H5 vaccine protects chickens and domestic ducks from lethal infection of the highly pathogenic H5N6 avian influenza virus. Front Vet Sci 8:773715. doi: 10.3389/fvets.2021.773715 35187138 PMC8850738

[ref15] Li H. Liu G. Zhou Q. Yang H. Zhou C. Kong W.. (2023). Which strain of the avian coronavirus vaccine will become the prevalent one in China next? Front Vet Sci 10:1139089. doi: 10.3389/fvets.2023.1139089, PMID: 37215473 PMC10196085

[ref16] Najimudeen S. Hassan M. S. Cork S. Abdul-Careem M. F. (2020). Infectious bronchitis coronavirus infection in chickens: multiple system disease with immune suppression. Pathogens 9:779. doi: 10.3390/pathogens9100779 32987684 PMC7598688

[ref17] Pereira C. G. Saraiva G. L. Vidigal P. M. Fietto J. L. Bressan G. C. Moreira M. A.. (2016). Distribution of infectious bronchitis virus strains in different organs and evidence of vertical transmission in natural infection. Arch. Virol. 161, 3355–3363. doi: 10.1007/s00705-016-3030-5, PMID: 27586414 PMC7087270

[ref18] Reed L. J. Muench H. (1938). A SIMPLE METHOD OF ESTIMATING FIFTY PER CENT ENDPOINTS12. Am. J. Epidemiol. 27, 493–497. doi: 10.1093/oxfordjournals.aje.a118408

[ref19] Rohaim M. A. El Naggar R. F. Abdelsabour M. A. Mohamed M. H. A. El-Sabagh I. M. Munir M. (2020). Evolutionary analysis of infectious bronchitis virus reveals marked genetic diversity and recombination events. Genes 11:60605. doi: 10.3390/genes11060605 PMC7348897 32486006

[ref20] Samy A. Naguib M. M. (2018). Avian respiratory coinfection and impact on avian influenza pathogenicity in domestic poultry: field and experimental findings. Vet Sci 5:10023. doi: 10.3390/vetsci5010023 PMC5876583 29495276

[ref21] Shirvani E. Paldurai A. Manoharan V. K. Varghese B. P. Samal S. K. (2018). A recombinant Newcastle disease virus (NDV) expressing S protein of infectious bronchitis virus (IBV) protects chickens against IBV and NDV. Sci. Rep. 8:11951. doi: 10.1038/s41598-018-30356-2, PMID: 30097608 PMC6086832

[ref22] Tan L. Liao Y. Fan J. Zhang Y. Mao X. Sun Y.. (2016a). Prediction and identification of novel IBV S1 protein derived CTL epitopes in chicken. Vaccine 34, 380–386. doi: 10.1016/j.vaccine.2015.11.042 26620841

[ref23] Tan L. Wen G. Qiu X. Yuan Y. Meng C. Sun Y.. (2019). A recombinant La Sota vaccine strain expressing multiple epitopes of infectious bronchitis virus (IBV) protects specific pathogen-free (SPF) chickens against IBV and NDV challenges. Vaccines 7:40170. doi: 10.3390/vaccines7040170 PMC6963182 31683905

[ref24] Tan L. Zhang Y. Liu F. Yuan Y. Zhan Y. Sun Y.. (2016b). Infectious bronchitis virus poly-epitope-based vaccine protects chickens from acute infection. Vaccine 34, 5209–5216. doi: 10.1016/j.vaccine.2016.09.022, PMID: 27665355

[ref25] Tcheou J. Raskin A. Singh G. Kawabata H. Bielak D. Sun W.. (2021). Safety and immunogenicity analysis of a Newcastle disease virus (NDV-HXP-S) expressing the spike protein of SARS-CoV-2 in Sprague Dawley rats. Front. Immunol. 12:791764. doi: 10.3389/fimmu.2021.791764, PMID: 34868082 PMC8637447

[ref26] Zhao R. Sun J. Qi T. Zhao W. Han Z. Yang X.. (2017). Recombinant Newcastle disease virus expressing the infectious bronchitis virus S1 gene protects chickens against Newcastle disease virus and infectious bronchitis virus challenge. Vaccine 35, 2435–2442. doi: 10.1016/j.vaccine.2017.03.045, PMID: 28342665

